# Supplementary material for: First-Line Use of Daratumumab in Patients with Multiple Myeloma Shows Delayed Neutrophil and Platelet Engraftment after Autologous Stem Cell Transplantation: Results from a Real-Life Single-Center Study
Source: Cancers (Basel). 2024 Sep 27;16(19):3307. doi: 10.3390/cancers16193307 (PMC11475878; doi:10.3390/cancers16193307)
Supplement: Supplementary file 1 [file cancers-16-03307-s001.zip › cancers-3192254-supplementary.pdf]

Table S1. multivariable logistic model

a. Multivariable logistic model on FN

	Sign.	OR	95% C.I.per EXP(B)	
			Inferiore	Superiore
Gender M vs F	.609	1.198	.599	2.397
Age at transplant	.374	1.021	.976	1.067
D-VTd versus VTd	.026	2.217	1.100	4.467
CD34+ $\geq$ 4 vs <4	.867	.937	.439	1.998

b. Multivariable logistic model on diarrhea

	Sign.	OR	95% C.I.per EXP(B)	
			Inferiore	Superiore
Gender M vs F	.371	.634	.234	1.720
Age at transplant	.404	1.029	.962	1.101
D-VTd versus VTd	<.001	8.317	2.568	26.939
CD34+ $\geq$ 4 vs <4	.422	.661	.241	1.816
